# MicroRNA Expression Changes in Kidney Transplant: Diagnostic Efficacy of miR-150-5p as Potential Rejection Biomarker, Pilot Study

**DOI:** 10.3390/jcm10132748

**Published:** 2021-06-22

**Authors:** Rafael Alfaro, Isabel Legaz, Victor Jimenez-Coll, Jaouad El kaaoui El band, Helios Martínez-Banaclocha, José Antonio Galián, Antonio Parrado, Anna Mrowiec, Carmen Botella, María Rosa Moya-Quiles, Francisco Boix, Jesús de la Peña-Moral, Alfredo Minguela, Santiago Llorente, Manuel Muro

**Affiliations:** 1 Immunology Service, University Clinical Hospital, Virgen de la Arrixaca-Biomedical Research Institute of Murcia (IMIB), 30120 Murcia, Spain; raf.hellin@gmail.com (R.A.); victorjcoll@gmail.com (V.J.-C.); jaouad88@hotmail.es (J.E.k.E.b.); heliosmar@live.com (H.M.-B.); diegoarmandogalian@hotmail.com (J.A.G.); antonio.parrado@carm.es (A.P.); aniamrowiec@gmail.com (A.M.); carmen.botellam@gmail.com (C.B.); rosa.moya2@carm.es (M.R.M.-Q.); franboix82@hotmail.com (F.B.); alfredo.minguela@carm.es (A.M.); 2 Department of Legal and Forensic Medicine, Faculty of Medicine, Biomedical Research Institute (IMIB), Regional Campus of International Excellence “Campus Mare Nostrum”, University of Murcia, 30100 Murcia, Spain; isalegaz@um.es; 3 Pathology Services, University Clinical Hospital, Virgen de la Arrixaca-Biomedical Research Institute of Murcia (IMIB), 30100 Murcia, Spain; jmpc1@um.es; 4 Nephrology Services, University Clinical Hospital, Virgen de la Arrixaca-Biomedical Research Institute of Murcia (IMIB), 30100 Murcia, Spain; sllorentev@telefonica.net

**Keywords:** miRNA PCR array, gene expression, kidney transplant, miRNA, rejection, forensic pathology

## Abstract

Background: The kidney allograft biopsy is considered the gold standard for rejection diagnosis but is invasive and could be indeterminate. Several publications point to the role of miRNA expression in suggesting its involvement in the acceptance or rejection of organ transplantation. This study aimed to analyze microRNAs involved in the differentiation and activation of B and T lymphocytes from kidney transplant (KT) patients’ peripheral blood leukocytes to be used as biomarkers of acute renal rejection (AR). Methods: A total of 15 KT patients with and without acute rejection (AR/NAR) were analyzed and quantified by miRNA PCR array. A total of 84 miRNAs related to lymphocyte differentiation and activation B and T were studied. The functions and biological pathways were analyzed to predict the potential targets of differential expressed miRNAs. Results: Six miRNA were increased in the AR group (miR-191-5p, miR-223-3p, miR-346, miR-423-5p, miR-574-3p, and miR-181d) and miR-150-5p was increased in the NAR group. In silico studies showed a total of 2603 target genes for the increased miRNAs in AR, while for the decrease miRNA, a total of 1107 target-potential genes were found. Conclusions: Our results show that KT with AR shows a decrease in miR-150-5p expression compared to NAR, suggesting that the decrease in miR-150-5p could be related to an increased MBD6 whose deregulation could have clinical consequences.

## 1. Introduction

The kidney allograft biopsy is considered the gold standard for rejection diagnosis [1]. However, the kidney biopsy is invasive and could be indeterminate [2]. Nevertheless, significant progress has been made in identifying new kidney rejection biomarkers, and some have been released recently for future use in clinical practice [3,4].

MicroRNAs (miRNAs) are non-coding RNA molecules, small, endogenous RNAs that inhibit translation of target mRNAs and are currently an active study area. It is estimated that about 60% of the human transcriptome is regulated by these molecules [5,6]. The recent development of high-throughput sequencing technologies [7] and predictive computational and bioinformatics methods have greatly enhanced miRNA research, including regulatory targets and possible functions [8]. Many miRNAs are known for their functions in various processes, including cell proliferation, cell death, fat metabolism, neuronal patterning, hematopoietic differentiation, immunity, heart transplant rejection, hematopoietic stem cell transplantation [9,10,11,12].

The expression of miRNAs in B lymphocytes has been studied in different subpopulations, such as germline naive B lymphocytes, showing that these molecules may play an essential role in cell development and effector functions [13,14]. For example, it has been observed that miR-150 deficient mice have increased antibody secretion after antigenic stimulation [15]. On the other hand, the defects in miR-155 produce a defect in isotype change and a poor differentiation towards plasma cells [16].

In recent years, several publications point to the role of miRNA expression in solid organ transplantation and suggest its involvement in the processes of acceptance or rejection of allograft [10,13]. Danger et al. [17] showed increased miR-142-3p in B lymphocytes from tolerant kidney transplant (KT) recipients, suggesting a possible mechanism involved in the TGF-β signaling pathway.

Furthermore, the study of miRNA expression profiles has demonstrated its usefulness for predicting kidney graft status in different clinical settings such as acute rejection or delayed graft function [18,19]. However, these data need to be confirmed in a more extensive patient series.

This study aimed to analyze 84 microRNAs involved in the differentiation and activation of B and T lymphocytes from KT patients’ peripheral blood to be used as biomarkers to diagnose acute rejection (AR).

## 2. Patients and Methods

### 2.1. Demographic Data and Clinical Characteristics

A total of 15 patients were recruited at the University Clinic Hospital “Virgen de la Arrixaca” (Murcia, Spain) and retrospectively analyzed. All patients had the same sociodemographic characteristics and equal treatment, exact time of the AR presence, equality age, and gender, consisting of 10 non-acute rejections (NAR) and five acute rejections (AR). In addition, no significant differences were observed regarding age, sex, HLA incompatibilities, and donor type between these selected patients (Table 1).

Estimated glomerular filtration rate (eGFR) and creatinine were analyzed in all transplant patients (normal values between brackets): creatinine (0.7 to 1.2 mg/dL) and eGFR (>90 mL/min/1.73 m^2^). The estimated glomerular filtration values were calculated based on the MDRD equation (Modification of Diet in Renal Disease). Our patients’ cohort showed the following values before the transplant; creatinine (mg/dL; 2.9 ± 2.1; mean ± SD) and an eGFR less than 60 mL/min/1.73 m^2^ more than three months suggests a chronic kidney disease [20,21,22,23].

Of all the patients analyzed, only patients whose kidney graft was in operation for at least one month post-transplantation and had monitoring for donor-specific antibodies (DSA) were included. Luminex determinations for detecting anti-HLA antibody screening before transplantation were included in this study. Allograft loss was estimated as a return to dialysis.

Two patients in the NAR group (20%) had anti-HLA non-DSA antibodies before transplantation, while one in the AR group (20%) had performed anti-HLA antibodies.

All NAR patients presented an excellent post-transplant evolution (understanding good evolution to involve the absence of DSAs and acute rejection, and EGF less than 60 mL/min/1.73 m^2^ at one year post-transplantation). The exclusion criteria were related to reasons that could affect gene expression levels, such as that none of the patients included had a bacterial or viral infection or had an active reactivation of CMV at the time of sampling.

All patients gave their informed consent for inclusion before they participated in the study. The study was conducted in accordance with the Declaration of Helsinki, and the Ethics Committee approved the protocol of HCUVA (PI15/01370 and PI19/01194).

### 2.2. Immunosuppressive Treatment

All enrolled recipients had similar triple immunosuppressive therapy, consisting of oral tacrolimus (Prograf, Astellas, Ireland), mycophenolatemofetil (MMF, CellCept, Roche, Switzerland), and prednisolone (Dacortin, Merck, Spain). Tacrolimus (FK) based protocol was started at the dose of (0.10–0.15 mg/kg/day) and the dose was adjusted to maintain a trough level of FK in whole blood between 8 and 12 ng/mL during the first month postoperatively, between 7 and 10 ng/mL during 2–3 months after transplant and between 5 and 8 ng/mL after that. MMF was started at the dose of 2000 mg/day, with the dose decreasing to 1000–1500 mg/day during the first month postoperatively, depending on white blood cell count.

Methylprednisolone was administered intravenously at doses of 500, 250, and 125 mg/day on the day of transplantation, day 1–2, and day 3–4 after the operation, respectively. Oral prednisolone was started on day five after the operation at the dose of 20 mg, then tapered to 5–10 mg/day within 2–3 months after transplant.

### 2.3. Kidney Rejection Diagnosis

Allograft acute cellular rejection (ACR) was defined as an increase in serum creatinine at least 20% above baseline serum creatinine and as a biopsy-proven rejection (specimens were evaluated by light microscopy and immunofluorescence staining with a marker of classical complement activation (C4d) and classified according to Banff classification as updated in 2017) [24]. The follow-up period of the patients was one year after transplantation without rejection or with rejection. During the follow-up period, monitoring of serum creatinine values and estimated glomerular filtration was carried out.

The diagnosis of acute antibody-mediated rejection (AMR) requires the presence of distinguishable histopathological findings, positive C4d staining in peritubular capillaries, and the simultaneous presence of DSA (donor-specific antibodies) [25]. For the kidney, a consensus was reached that a diagnosis of AMR requires the simultaneous presence of DSA, distinguishable histopathological findings, and deposition of C4d in peritubular capillaries.

Mild acute cellular rejection (Banff grade I) was treated with pulse steroids (500 mg methylprednisolone boluses) and increased maintenance immunosuppression. All other ACR were treated with anti-thymocyteglobulin (ATG).

AMR was also treated with pulse steroids and intravenous immunoglobulin (0.25 g/kg and the last session 1 g/kg (maximum 140 g) was divided into two doses associated with plasmapheresis (three sessions a day, every five days). Later, we administered 500 mg anti-CD20 (Rituximab, Roche pharmaceuticals) intravenously. Anti-AMR treatment was also administered in two patients receiving anti-proteasome inhibitor Bortezomib (Velcade^®^, formerly PS-341).

### 2.4. Total RNA Extraction

Peripheral blood (PB) patients samples were collected on the same day of kidney rejection for miRNA expression profile analysis. Then, blood samples were lysed with Cell Lysis Solution Genomic Purification (Promega, Madison, WI, USA).

Peripheral blood leukocytes were separated from freshly isolated venous blood by centrifugation and washing. miRNA was isolated using the Maxwell 16 miRNA Kit (Promega, Madison, WI, USA) in a Maxwell 16 kit (Promega, Madison, WI, USA) according to the instructions. The RNA concentration and its purity were measured using NanoDrop2000 (ThermoScientific, Wilmington, CA, USA). Pure RNA samples were considered, with ratios between 2.0 and 2.2 spectrophotometric measurements of absorbance at 260/280 nm light length.

### 2.5. RT-PCR for miRNAs

miRNA was reverse-transcribed to cDNA using the miScript II RT kit (Qiagen, Hilden, Germany) following the manufacturer’s recommendations. Briefly, 500 ng of total RNA was mixed with 4 µL of miScriptHiSpec Buffer, 2 µL of miScriptNucleics Mix, 2 µL of miScript Reverse Transcriptase Mix, and RNase-free water to a final volume of 20 µL. Finally, the mixture was incubated for one hour at 37 °C and then five minutes at 95 °C to stop the reaction. cDNAs were next diluted in nuclease-free water before qPCR reactions. Subsequently, the cDNA samples were stored at −20 °C until the moment of use.

### 2.6. miRNA Expression Profile Analysis

Finally, for the miRNA expression profile analysis, the SYBR Green-based qPCR methodology used the miScriptmiRNA PCR Array Human T-Cell and B-Cell Activation panel (Qiagen, Las Matas, MD, Spain) that allows the quantification of 84 miRNAs related to B and T lymphocyte differentiation and activation.

miRNAs included in the panel are shown in Table 2, and these were differentially expressed depending on whether they were with or without acute rejection development. Reactions for qPCR were carried out in special 96-well plates on an ABI-7500 Fast Real-Time PCR System kit (Applied Biosystem, BV, Singapore). Each reaction was performed twice, and the arithmetic mean of these two measures was taken for further analysis.

A total of 500 ng of the cDNA was mixed with 1375 µL of QuantiTect SYBR Green PCR Master Mix (Qiagen, Hilden, Germany), 275 µL of miScript Universal Primer, and RNase-free water to a final volume of 2750 µL. Next, 25 µL of the above mixture was added to each well of the plate. The thermal cycler conditions microarray was for Taq polymerase 15 min 95 °C (initial activation) and 40 cycles of 94 °C 15 s, 55 °C 30 s, and 70 °C 30 s. This method was used to identify miRNAs differentially expressed (DE) in 10 transplant samples with stable function during the first post-transplant year (NAR) and five transplant samples with AR.

To normalize RT-PCR data, the average value obtained from six control genes was used that the array carried used (SNORD61, SNORD68, SNORD72, SNORD95, SNORD96A, and RNU6-2) as recommended by the manufacturer.

miRNA expression was then normalized to 28 S rRNA expression that was very stable in all samples. miRNAs were considered differentially expressed with a Fold-change (Fch > 2) and Cycle threshold (Ct) value < 35. The Ct value is the number of PCR cycles for a given miRNA at which the fluorescence signal exceeds the established threshold. A Ct value less than 35 was used to establish the presence or absence of a miRNA as recommended by the manufacturer. miRNAs that met these criteria were chosen for subsequent bioinformatics analyses. RNase-free water was used as a negative control.

### 2.7. Target Prediction of miRNAs Differentially Expressed

Two bioinformatics tools were used to predict miRNAs’ potential target genes differentially expressed (DE) between AR and NAR. The algorithms DIANA-microT-CDS [26] and TargetScan v7.1 [27] were used. For the DIANA-microT-CDS algorithm, a cut-off value of 0.7 was used. For the selection of targets in TargetScan v7.1, the first 200 predicted targets were used. Only the target genes predicted by both algorithms were taken into account for subsequent analysis, increasing the prediction’s sensitivity. Both applications, DIANA, and TargetScan were used in this study. In any case, the study always focused on experimentally demonstrated genes.

These computational methods use complementary sequence base-pairing between miRNA and mRNA in target prediction. Files and databases containing miRNA and predicted target genes were downloaded from the online websites of these toolsets. Four cohorts of kidney transplants (KTs) (GSE115816, GSE14346, GSE15296, and GSE46474) were used to compare target genes’ expression levels in whole peripheral blood (Supplementary Table S1) [28,29,30,31].

### 2.8. Functional Analysis of miRNAs Differentially Expressed in Acute Rejection

miRNAs were considered differentially expressed, being the cut-off established with a Fold-change (Fch > 2) and Ct value < 35 in AR and NAR group. Fold-change positive values indicate an increase in the AR group, and negative values indicate decreased NAR.

miRNAs differentially expressed between AR and NAR were functionally analyzed by GO terms [32] to know the biological processes (BP) and molecular function (FM). For the terms analysis, only those related to molecular function and biological processes were considered. GO terms with an adjusted *p*-value < 0.05 were considered significant.

To know the major biological pathway-based target gene, Kyoto Genomes and Genomes Encyclopedia (KEGG) was used [33]. KEGG-pathways are a collection of genomes/genomes database, enzyme pathways, and the biological processes that infer the major biological pathways affected by a set of given genes. First, the minimum number of genes to be considered a path was adjusted to a value of two. Next, the *p*-value obtained from each biological route was adjusted by Benjamini and Hochberg’s FDR method [34]. Biological pathways with FDR < 0.05 were considered significant.

### 2.9. Validation of miRNAs in the GSE115816 Cohort

We performed a pilot validation of these miRNAs differentially expressed in the AR group. The raw gene expression data from the mRNA microarray data were available in the Gene Expression Omnibus [35] with accession no. GSE115816. The GSE115816 study was performed on an Illumina HiSeq 2500 platform. It consisted of six samples of total RNA extracted from leukocytes from control peripheral blood with good renal function and 10 recipients with AR (six with antibody-mediated rejection (AMR) and four with cell-type rejection) [36].

### 2.10. Statistical Analysis

Demographic data and results were collected in a database (Microsoft Access 2.0; Microsoft Corporation, Seattle, WA, USA) and the analysis was performed using SPSS 23.0 (SPSS software Inc., Chicago, IL, USA). All results were expressed as the mean ± SD or as a percentage. Demographic, clinical, immunological features, and post-transplant anti-HLA antibodies status were compared using Pearson χ^2^ test or Fisher’s exact test for categorical data and Student *t*-test or Mann–Whitney U test for continuous data, as appropriate. A two-sided *p*-value < 0.05 was considered statistically significant.

## 3. Results

### 3.1. miRNA Expression Profile Related to the Differentiation and Activation of B and T Cells in Kidney Recipients’ Outcome

**Figure 1:**
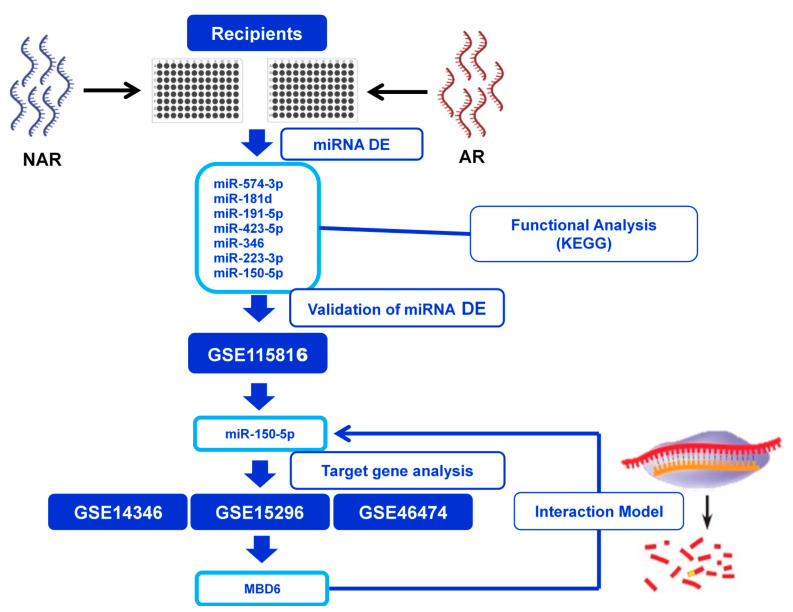
Design of the miRNA expressed study in kidney transplantation outcome. The study was divided into four stages: (1) Identification of differentially expressed miRNAs between transplanted without rejection (NAR) and acute rejection (AR). (2) Functional analysis to discover the main biological pathways affected by DE miRNAs. (3) Validation of DE miRNAs in an independent cohort obtained from the GEO database. (4) Identification of the validated miRNAs’ target genes and evaluation in three gene expression cohorts to subsequently establish a hypothesis of a target gene-miRNA interaction model.

As shown in Figure 1 and Table 2, a total of 84 miRNA involved in the differentiation and activation of B and T lymphocytes were analyzed to identify the differentially expressed miRNAs between the NAR and AR groups. miRNA was considered when Ct < 35 in at least one of the compared groups. The detected miRNAs are shown in Table 3 and Table 4. In our cohort, 76 miRNAs (90.5%) included in the protocol were detected.

In the NAR group, a total of 53 miRNAs (63.1%) were detected, whereas, in the sample AR group, there were 75 miRNAs (89.3%). As shown in Table 3, of the total of 84 miRNAs studied, seven differentially expressed miRNAs were found in AR (miR-181d, miR-191-5p, miR-223-3p, miR-346, miR-423-5p, miR-574-3p) and miR-150-5pin the NAR group. Within the increased miRNAs in the AR group, the most remarkable differences were found in miR-574-3p (Fch: 28.40), miR-181d (Fch: 22.54), and miR-191-5p (Fch: 13.68).

### 3.2. Target Genes of miRNAs Differentially Expressed

To know the target genes for miRNA, in silico analysis was performed using the DIANA-microT-CDS v5.0 and TargetScan v7.1 algorithm. For the increased miRNAs (miR-574-3p, miR-181d, and miR-191-5p) in KT recipients of the AR group, a total of 2603 target genes were observed, while for the decreased miRNA a total of 1107 target-potential genes were found.

### 3.3. Biological Processes of Target Genes of miRNA Differentially Expressed in Kidney Transplantation

The biological processes (BP) were analyzed in the target genes of miRNA differentially expressed in the KT with AR (Table 4).

Regarding the target genes of miRNA decreased in the AR group, the functional analysis GO (Table 4B) was also involved in 5 BP and five different MF. The main biological processes were related to the differentiation of cellular myeloid (FDR = 0.0019) and cell–cell adhesion via membrane proteins (FDR = 0.0033). MF is usually related to the binding of DNA and RNA (FDR < 0.0001).

### 3.4. Molecular Functions of Target Genes of miRNA Differentially Expressed in Kidney Transplantation

KEGG analyzed the MFs in miRNA target genes differentially expressed in the KT with AR (Table 5). Regarding miRNA increase, five differentially expressed routes were obtained (Table 5A). Of all of them, the signaling route of cAMP (FDR = 0.0039) and the regulation of phosphatidyl-inositol signaling (FDR = 0.0040) stand out due to their relevance in immune activation.

Concerning miRNA decreased (Table 5B), showed two differentially expressed pathways, ErbB signaling pathways (FDR = 0.007) and resistance to Epidermal Growth Factor Receptor (EGFR) tyrosine kinase inhibitors (FDR = 0.026).

### 3.5. Validation of miRNAs in the GSE115816 Cohort

**Figure 2:**
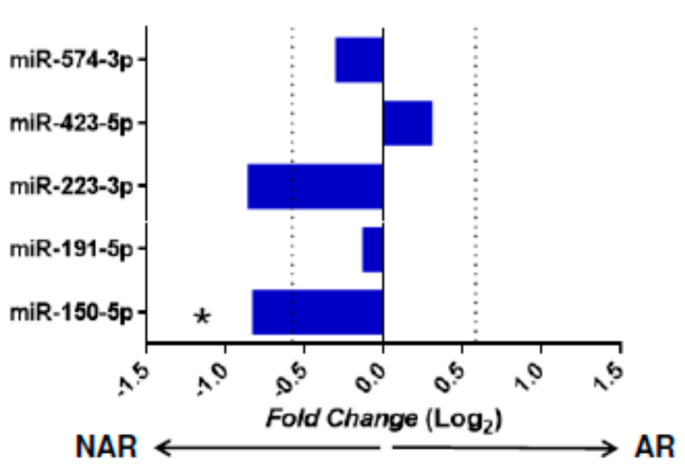
Expression of miRNA in the GSE115816 cohort. Validation of miRNAs with statistical significance in the independent GSE115816 cohort (*n* = 16). The graph represents the receptor miRNAs’ Fold-change values without acute rejection (NAR, *n* = 6) and acute rejection (AR, *n* = 10). Comparisons were made using the Mann–Whitney U test. miRNAs with a value of *p* < 0.05 and a Foldchange (Log2) > 0.58 were considered significant. The cut-off value of the fold-change is indicated with a vertical dotted line. * *p* < 0.05, arrows indicate the group in which the miRNA is increased.

The raw gene expression data from study GSE115816 (downloaded from the public GEO database) were used to validate miRNAs with differential expression (DE) in our KT patients. miR-346 and miR-181d were not evaluated as they were not included in the miRNA panel of this study. The Matz et al. [37] (*p* < 0.05 and an absolute F old-change [Log2] > 0.58) criteria were used to consider a statistically significant miRNA. When comparing our results with the GSE115816 cohort, a significant decrease in miR-150-5p gene expression (*p* = 0.031, Fold-change [Log2] = 0.82) was observed in the AR group confirming the results obtained in our study. For the rest of the miRNAs evaluated (miR-191-5p, miR-423-5p, and miR-574-3p), no significant differences were obtained (Figure 2).

Therefore, to rule out possible bias in our study sample selection, we compared the absolute lymphocyte levels of the peripheral blood samples from which the RNA used was isolated. The results indicated that although in the kidney transplant rejection (KTRs) with AR they had fewer lymphocytes with respect to NAR, the differences were not significant (1852 ± 1025 cell/µL vs. 1025 ± 667 cell/µL; *p* = 0.188), so the decrease in miR-150-5p should be a consequence of a clinical event and not due to differences in the blood count.

### 3.6. miR-150-5p Target Genes and Expression Analysis in Transcriptomic Studies

Our results showed that miR-150-5p was deregulated in KT patients with AR and also in the cohort GSE115816. For this reason, miR-150-5ptarget genes and their expression were analyzed. miR-150-5ptarget genes were searched using the DIANA-microT-CDS v5.0 tool, and three cohorts of KTs (GSE14346, GSE15296, and GSE46474) with the levels of expression of mi-150-5p target genes in peripheral blood (Table 6).

Supplementary Table S2 shows the comparisons, in each cohort, of the expression of the genes in which MBD6 interacts between patients with rejection (AR) and without rejection (NAR). The four significant genes (HCFC1, SETD1A, ASH2L, and OGT) indicate an increase in the expression of these genes in the NAR group. However, as they have been significant in one cohort but not in the others, it can be concluded that they are not genes that are altered in a generalized way in rejection events. In the others, there are no differences between the NAR and AR groups.

The decrease in the gene expression of miR-150-5p in KTR with AR could indicate a variation in the genetic expression (increase or decrease) of target genes.

A total of 22 possible target genes were found, and only Methyl-CpG Binding Domain Protein 6 (MBD6) was increased in the three cohorts analyzed. MBD6 was significantly increased in the GSE14346 cohort (*p* < 0.001, FDR = 0.006), GSE15296 cohort (*p* = 0.003, FDR = 0.022) and GSE46474 (*p* = 0.020, FDR = 0.308), although in the latter it loses its significance when adjusting for multiple comparisons.

These data suggest a possible miR-150-5p/MBD6 interaction during kidney AR. During AR events, miR-150-5p levels decrease, probably causing an increase in MBD6 expression levels and provoking transcriptional changes that promote the activation and/or proliferation of the immune system’s cells.

**Figure 3:**
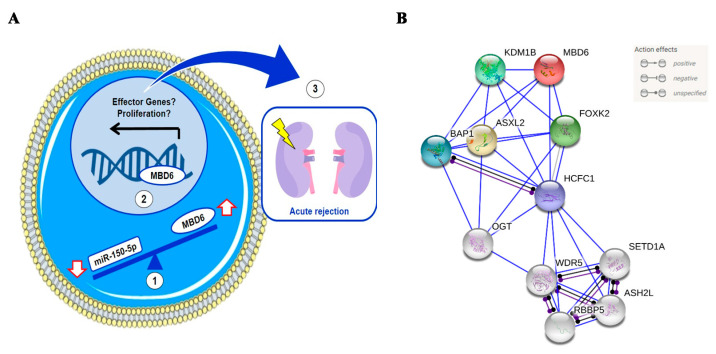
(**A**) Hypothetical miR-150-5p and MBD6 interaction model in leukocytes from peripheral blood. (1) According to our hypothesis, the decrease in the expression of miR-150-5p in leukocytes from peripheral blood causes an increase in the expression of MBD6. (2) The increase in MBD6 causes an alteration in the gene expression patterns, perhaps promoting the expression of effector genes or cell proliferation. (3) The expression of effector and/or proliferation genes promotes the immune system’s attack on renal graft and acute rejection. (**B**) Analysis of protein-protein interactions of MBDA and predicted functional partners. Red bubble, MBDA; yellow, ASXL2, dark green, FOXK2; light green, KDM1B; blue, BAP1; Purple, HCFC1.

Besides, a protein–protein interaction analysis was performed to observe the interaction of the MBD6 protein with other proteins (Figure 3B). The study showed that MBDA protein interacted with five proteins (KDM1B, BAP1, ASXL2, HCFC1, FOXF2). KDM1B is an epigenetic mark that regulates gene expression and chromatin function [38]. BAP1 is a protein that functions as a deubiquitinase, which removes a molecule called ubiquitin from specific proteins. ASXL2 is involved in the assembly of transcription factors at specific genomic loci. Finally, HCFC1and FOXF2 are both related to DNA-binding transcription factor activity and chromatin binding.

## 4. Discussion

In this study, a total of 84 microRNAs involved in the differentiation and activation of B and T lymphocytes from KT patients’ peripheral blood were analyzed to study the differences in the kidney transplant outcome and how they can be used as AR biomarkers in peripheral blood.

Since their discovery, miRNAs have been proposed as potential biomarkers for diagnosing and treating diseases and other clinical situations such as allograft rejection [39,40]. In our study, AR KT-patients revealed an increase of six miRNAs (miR-191-5p, miR-223-3p, miR-346, miR-423-5p, miR-574-3p, and miR-181d) and a decrease of miR-150-5p expression. Subsequent analyses showed a possible interaction between miR-150-5p and transcription factor MBD6, suggesting that this interaction’s alteration may contribute to AR episodes’ initiation or development.

miR-150 is an essential miRNA in immune regulatory functions, such as proliferation, activation, and apoptosis of B and T lymphocytes, and is selectively expressed in lymph nodes, spleen, and mature B T-cells [14,41]. It is involved in innate and adaptive immune responses, as it is one of the most studied miRNAs and plays an essential role in some tumors’ pathogenesis [42,43,44].

This miRNA is involved in Tregs cell differentiation through the regulation of mTOR expression [45]. In this sense, miR-150 has been proposed as a marker of lymphocyte activation [46]. While a decrease in miR-150-5p expression in KTRs with AR was observed in our cohort, other similar studies observed an elevation of miR-150 in patients with ACR compared to biopsies without rejection [47]. However, other studies did not observe differences in biopsies or peripheral blood mononuclear cells [48]. Furthermore, Candia et al. [46], after activating CD4+ cells, a decrease of miR-150 occurs at the intracellular level correlated with increased serum. These observations have been corroborated in autoimmune diseases [49]. A study [46] suggested that the release of miR-150 to the extracellular medium within nanoparticles may represent a new mechanism for regulating the intracellular levels of miR-150, the expression of genes that it targets, and that are critical for immune activation. This is compatible with our observations, where the activation of CD4+ cells could be associated with the decrease in miR-150-5p. Although serum levels have not been measured in our work, in murine pancreatic transplant models, an elevation of plasma miR-150-5p was associated with graft rejection and destruction of β cells [50].

In our study, miR-181d also showed a 22.5-fold increase in AR compared to NAR patients, where it did not. The miR-181d is one of the four members of the miR-181 family and has been associated with tumoral pathologies [51]. The decrease in its expression has been associated with acute renal damage in murine models due to ischemia-reperfusion [52]. Although other miRNAs from the miR-181 family, such as miR-181a, have been associated with AR in KT, there are no data on the implication of miR-181d [53]. It is involved in the differentiation and activation of B and T cell development.

On the other hand, miR-574-3p was the miRNA with the most considerable difference (~28.4-fold increase) between KTRs and without AR. Our KT study observed that this miRNA’s increase was associated with delayed graft function patients, although the results were not subsequently confirmed in a validation cohort [19]. In other studies, miR-574 was decreased in liver recipients suffering AR, although these differences were not observed compared with biopsies with AMR [47]. Yang et al. [54] showed that miR-574-3p negatively modulates the IL-6/STAT3 pathway. Thus, the decrease in miR-574-3p expression in samples with T cell-mediated rejection [47] could increase pro-inflammatory pathways derived from STAT3 activation [54].

Analysis of the biological pathways of KEGG indicates that the increased miRNAs in AR target genes are involved in cAMP and phosphatidylinositol signaling pathways. Regarding miR-150-5p, the only miRNA with a significant decrease in the group with AR, two over-represented biological pathways were obtained, the ErbB signaling pathway and resistance to EGFR inhibitors.

A search in the GEO database of miRNA gene expression in KTRs was performed to confirm our results. From the previous search, we obtained study GSE115816 belonging to Matz et al. [37], which used the Illumina HiSeq 2500 platform for miRNA detection. We focused the search for miRNA biomarkers of AMR and interstitial fibrosis/tubular atrophy. In our study, miRNA expression data from the GSE115816 cohort were re-analyzed, establishing two groups, AR and NAR.

Our results showed that miR-150-5p was the only miRNA that showed significant differences in our cohort and the GSE115816 cohort. However, failure to obtain differences in the other miRNAs does not exclude their AR involvement since it must be considered that the GSE115816 cohort had only 16 samples, and the different quantification methods (RT-PCR in our cohort against sequencing massive in GSE115816) could introduce some differences.

The decrease in the expression of miR-150-5p in KTRs with AR implies that there must be a set of target genes whose expression is reciprocally increased. That is why we decided to study the experimentally validated targets of miR-150-5p in different KTR cohorts to discover some miRNA-gene interactions that could potentially be involved in AR.

The three cohorts analyzed the MBD6 gene increased in the three analyzed transcriptomic studies, suggesting a possible miR-150-5p/MBD6 interaction. In addition, this fact is also observed in our results, since in our cohort, it is observed that miR-150-5p levels decrease in the AR development group, and there is an increase in MBD6 expression levels, whose relationship should be analyzed in the future.

The MBD6 protein is a protein that binds to methylated DNA and is involved in processes of aging, proliferation, and cell survival by regulating the activity of Oct4 [55]. The role of MBD6 in the regulation of immune system cells has not been studied in depth yet. In B lymphocytes, stimulation with BAFF causes a decrease in the expression of MBD6 [56]. The action of MBD6, together with FOXK2, is vital in the recruitment of transcription factors so that the alteration of its expression through miRNAs can have important repercussions at the cellular level.

Future studies will be necessary to understand the actual relevance of miR-150-5p in acute renal graft rejection and its possible interaction with MBD6. Despite the novel results obtained, we have also had some limitations. Our study’s main limitation is the limited sample size, perhaps resulting in a loss of statistical significance in some of our analyses. Given the results obtained in this pilot study, it will advance the deepening and expansion of the sample size in the future. It should be noted that the study by Matz et al., 2018, carried out with a reduced sample size (*n* = 17), showed a decrease in miR-150-5p in patients with rejection, which supports our results, despite the sample size of our preliminary study. Although the results in miR-150-5p have been validated in a small cohort, it is necessary to validate them in larger groups in the future. Besides, it should be noted that in our study, miRNAs that were detectable in only one group were excluded from further analysis. However, these miRNAs could also be interesting and provide important information. Our study is a pilot study on a limited cohort of patients where no previous differences were observed between patients with different types of rejection. Our study aimed to analyze 84 microRNAs involved in the differentiation and activation of B and T lymphocytes from KT patients’ peripheral blood to be used as biomarkers to diagnose AR, regardless of the type of rejection.

It should be noted that the main objective of our work was to search for use as biomarkers of acute renal rejection (AR) regardless of the type of rejection. No previous differences were observed between patients with different types of rejection. Given the results obtained in this study, our research team will analyze larger cohorts in the future where it is possible to disaggregate the study population’s different characteristics.

The GEO databases extract RNA from whole blood, unlike our study where the RNA comes from leukocytes. miRNA expression profiles differ according to cell lineage. Therefore, obtaining RNA from different sources can generate different expression profiles. However, when comparing our results with those of Matz et al., [37], you can assume that miRNAs from sources other than leukocytes, such as erythrocytes and platelets, would not significantly affect the miRNA comparisons we use in our panel as these are found at very low levels in enucleated cells (erythrocytes and platelets). For example, according to the work of Teruel-Montoya et al., [57], only 1% of the total miR-150-5p obtained from whole blood comes from cellular sources other than leukocytes (it comes mainly from lymphocytes).

Similarly, miR-223-3p (also deregulated) comes almost exclusively from granulocytes [57,58]. Although erythrocytes have a wide richness of miRNA [59], given that these are enucleated cells, it can be assumed that the variations in the levels of gene expression found between study groups (both in miRNA studies and transcriptomic in general) are mainly due to the nucleated fraction of the blood (leukocytes). Therefore, it is expected that the changes observed in whole blood will be similar to those observed in RNA extracted from other sources such as leukocytes or lymphocytes. Möhnle et al., [60]. Observed that the decrease in miR-150 as a sepsis biomarker was deregulated in the same direction both in whole blood samples and in isolated CD4 T lymphocytes, which shows that specific miRNAs, such as miR-150-5p, are comparable results obtained from whole blood and leukocytes.

Finally, the miRNA target databases are mostly made up of theoretical interactions that are not experimentally validated, making an honest interpretation difficult. Although when studying the objectives of miR-150-5p, we only included those demonstrated, it must be taken into account that most have been carried out on cell cultures or on non-leukocyte cell types, which is why in future studies, it is necessary to validate these interactions on the type of sample used. Despite all these limitations, statistically robust analyses with promising diagnostic implications have been achieved in this study.

In conclusion, the results of this pilot study show that KT patients with AR show a decrease in miR-150-5p expression compared to NAR transplants, suggesting that a hypothetical decrease in miR-150-5p could be related to an increase of MBD6 whose dysregulation could have clinical consequences. Our findings suggest miR-150-5p as potential use of miRNAs to diagnose AR in peripheral blood of KT. Future studies should be performed to corroborate these results and be applied in clinical practice to avoid invasive kidney biopsy.

## Figures and Tables

**Table 1:** Demographic and clinical characteristics of kidney transplant patients whose miRNA expression profiles were analyzed.

Characteristics of Kidney Transplant Patients	Total *n* = 15	AR, *n* (%) *n* = 5 (33.3)	NAR, *n* (%) *n* = 10 (66.6)	*p* ^a^
Age receptor (years)	57.5 ± 9.15	61.2 ± 6.71	53.8 ± 11.6	0.371
Gender receptor (Male/Female), *n* (%)	15	3 (60)/2 (40)	6 (60)/4 (40)	1.000
Creatinine (v.n. 0.7 to 1.2 mg/dL)	2.9 ± 2.1	2.5 ± 1.7	1.9 ± 2.4	0.628
eGFR (v.n. >90 mL/min/1.73 m^2^)	<60 mL/min/1.73 m^2^	37 mL/min/1.73 m^2^	41 mL/min/1.73 m^2^	
HLA mismatches ^b^	6.2 ± 0.8	4.5 ± 0.64	4.0 ± 1.1	0.539
Live-Donor, *n* (%)	3	1 (20)	2 (20)	1.000
Preformed anti-HLA antibodies, *n* (%)	3	1 (20)	2 (20)	1.000
Induction therapy (Tim/Bas), *n* (%)	4	3 (60)/0 (0)	1 (10)/1 (10)	0.109
Delayed-Renal Function, *n* (%)	2	2 (40)	0 (0)	0.095

*n*, number of individuals with a particular disease; AR, Acute rejection; NRA, No Acute Rejection; Bas, Basiliximab; Tim, Thymoglobulin. eGFR, estimated glomerular filtration rate; v.n., Normal values. Quantitative data expressed as the mean ± standard error (SEM) of the mean. ^a^ Comparisons were made using Fisher’s exact test or χ^2^ for qualitative variables and the non-parametric U Mann–Whitney test for quantitative variables. *p* < 0.05 was statistically significant. ^b^ Total differences between donor and recipient concerning HLA-A, -B, and -DRB1 genes.

**Table 2:** Analysis of the differential expression of miRNAs included in the miScript miRNA PCR Array panel as a function of acute rejection in kidney recipients.

miRNA	NAR	Ct	AR	Ct	miRNA	NAR	Ct	AR	Ct
let-7a-5p	+	26.49	+	26.71	miR-19a-3p	-	37.48	-	37.25
let-7b-5p	+	28.43	+	28.96	miR-19b-3p	-	35.41	+	34.33
let-7c	+	30.38	+	30.78	miR-204-5p	-	35.89	+	33.99
let-7d-5p	+	28.66	+	28.77	miR-20a-5p	+	32.68	+	32.72
let-7e-5p	+	30.37	+	31.35	miR-20b-5p	+	34.69	+	33.56
let-7f-5p	+	29.52	+	29.84	miR-21-5p	+	30.53	+	30.71
let-7g-5p	+	30.26	+	30.51	miR-210	-	35.57	+	33.09
let-7i-5p	+	31.43	+	31.61	miR-214-3p	+	34.27	+	32.11
miR-100-5p	-	>40	-	>40	miR-221-3p	+	32.39	+	31.5
miR-101-3p	-	35.80	+	33.85	miR-222-3p	+	33.34	+	31.26
miR-106b-5p	+	33.85	+	30.93	miR-223-3p	+	28.1	+	26.9
miR-125b-5p	-	>40	+	33.53	miR-23a-3p	+	28.22	+	27.71
miR-126-3p	+	31.94	+	30.80	miR-23b-3p	+	32.2	+	30.95
miR-128	-	40.27	+	34.54	miR-24-3p	+	31.62	+	31.08
miR-130b-3p	+	34.31	+	31.35	miR-25-3p	+	30.16	+	29.99
miR-132-3p	-	35.44	+	34.89	miR-26a-5p	+	28.93	+	29.04
miR-139-5p	-	35.70	+	34.92	miR-26b-5p	+	29.23	+	29.08
miR-142-3p	+	34.54	+	33.51	miR-27a-3p	+	33.72	+	33.11
miR-142-5p	-	>40	-	35.03	miR-27b-3p	-	36.22	+	34.86
miR-145-5p	+	34.69	+	31.52	miR-28-5p	-	35.4	+	32.18
miR-146a-5p	+	33.04	-	35.47	miR-29a-3p	+	30.05	+	29.78
miR-146b-5p	-	>40	-	36.42	miR-29b-3p	-	37.14	+	33.8
miR-147a	-	>40	+	33.64	miR-29c-3p	+	32.97	+	32.52
miR-148a-3p	-	>40	+	32.72	miR-30a-5p	+	33.66	+	32.95
miR-150-5p	+	29.66	+	30.98	miR-30b-5p	-	36.18	+	32.62
miR-155-5p	+	30.01	+	30.34	miR-30c-5p	+	34.78	+	33.0
miR-15a-5p	+	34.55	+	34.32	miR-30d-5p	+	34.56	+	34.05
miR-15a-3p	-	35.15	+	32.2	miR-30e-5p	-	36.8	+	34.29
miR-15b-5p	+	27.26	+	26.58	miR-31-5p	+	34.46	+	33.34
miR-16-5p	+	30.51	+	30.32	miR-326	-	35.08	+	33.89
miR-17-5p	+	33.56	+	32.61	miR-331-3p	+	33.18	+	33.35
miR-17-3p	-	>40	+	33.61	miR-335-5p	-	39.15	-	35.31
miR-181a-5p	-	36.36	+	33.58	miR-342-3p	+	30.99	+	31.38
miR-181b-5p	+	30.67	+	30.94	miR-346	+	32.3	+	29.30
miR-181c-5p	-	35.45	+	33.61	miR-34a-5p	-	>40	-	35.64
miR-181d	+	33.48	+	28.85	miR-365a-3p	-	>40	-	35.42
miR-182-5p	+	33.79	+	32.32	miR-423-5p	+	30.32	+	27.27
miR-184	-	>40	-	35.4	miR-574-3p	+	32.72	+	27.76
miR-18a-5p	-	>40	-	35.0	miR-92a-3p	+	29.74	+	29.34
miR-191-5p	+	30.88	+	26.97	miR-93-5p	+	32.43	+	31.3
miR-195-5p	+	31.53	+	30.89	miR-98-5p	+	32.85	+	33.83
miR-199a-5p	-	>40	+	34.19	miR-99a-5p	-	>40	+	34.66

NAR. No acute rejection; AR. Acute rejection; +. miARN detected; -. miARN not detected.

**Table 3:** Analysis of differential expression miRNAs involved in the differentiation and activation of B and T lymphocytes of kidney transplant with or without acute rejection.

miARN	Fold-Change ^a^	Ct Value	
		AR	NAR
miR-574-3p	28.40	**27.76**	32.72
miR-181d	22.54	**28.85**	33.48
miR-191-5p	13.68	**26.97**	30.88
miR-423-5p	7.52	**27.27**	30.32
miR-346	7.28	**29.30**	32.30
miR-223-3p	2.09	**26.90**	28.10
miR-150-5p	−2.75	30.98	**29.66**

^a^ Cut-off was established with a Fold-change > 2 and a value of Ct < 35 in the AR and NAR group. Fold-change positive values indicate an increase in the AR group, and negative values indicate an increase in the NAR group. Values of Ct in bold indicate an increased miRNA.

**Table 4:** Function analysis of target genes of the miRNA decreased or increased in the AR group differentially expressed studied by Genetic Ontology (GO).

**A. Target Genes of the miRNA Increased in the AR Group**		
GO ID *	**Term ^a^**	**FDR ^b^**
**Biological process (BP)**		
GO: 0044265	Catabolic process of cellular macromolecules	0.0046
GO: 0016071	Metabolic process of mRNA	0.0046
GO: 0006401	Catabolic process of RNA	0.0046
GO: 0051150	Regulation of smooth muscle differentiation	0.0131
GO: 0006402	Catabolic process of thee mRNA	0.0236
**Molecular Function (MF)**		
GO: 0003723	Binding to RNA	0.0011
GO: 0032553	Binding to ribonucleotides	0.0018
GO: 0035639	Binding to ribonucleotide triphospates of purines	0.0018
GO: 0005524	Binding to ATP	0.0019
GO: 0008144	Binding to drugs	0.0020
**B. Target Genes of the Mirna Decreased in the AR Group**		
**Biological process**		
GO: 0061564	Axonal development	0.0001
GO: 0022604	Regulation of cell morphogenesis	0.0010
GO: 0016569	Covalent modification of chromatin	0.0019
GO: 0030099	Myeloid cellular differentiation	0.0019
GO: 0098742	Cell-cell adhesion via molecules membrane adhesion	0.0033
**Molecular Function**		
GO: 0003700	DNA binding transcription factor activity	<0.0001
GO: 0000981	DNA binding activity of RNA polymerase II	<0.0001
GO: 0043565	Binding to specific DNA sequences	<0.0001
GO: 0000976	Binding to specific DNA sequences of regulatory regions	<0.0001
GO: 1998037	Binding to specific sequences of double-stranded DNA	<0.0001

* GO ID, Genetic Ontology Identification; ^a^ Term, Function analysis of target genes; FDR. False Discovery Rate; BP. Biological process; MF. Molecular Function. Only the five most significant terms for each category (BP and MF) are shown. ^b^ FDR values < 0.05 were considered statistically significant. The functional analysis of the increased miRNA target genes in the AR group involved five BPs and five different molecular functions (MF). Concerning MF, most were involved in binding functions with other biomolecules and drugs (Table 4A).

**Table 5:** Biological pathway analysis of miRNAs differentially expressed in KT rejection.

**A. Target Genes of the miRNA Increased in the AR Group**		
**Route ID**	Term *	**FDR ^a^**
hsa04928	Secretion and synthesis of parathyroid hormone	0.0038
hsa04728	Dopaminergic synapsis	0.0038
hsa04371	Route of APLN signaling	0.0038
hsa04024	Route of cGMP signaling	0.0039
hsa04070	Route of phosphatidyl-inositol signaling	0.0040
**B. Target Genes of the miRNA Decreased in the Ar Group**		
hsa04012	Route of ErbBsignaling	0.0070
hsa01521	Resistance to TyrosinKinase EGFR	0.0260

APLN, Apelin; EGFR, Epidermal Growth Factor Receptor; FDR, False Discovery Rate. KT, Kidney transplantation; KEGG, Kyoto Encyclopedia of Genes and Genomes. * Biological pathways were analyzed using KEGG. ^a^ Values of FDR < 0.05 were considered statistically significant.

**Table 6:** Relationships of target genes found of the miR-150-5p in three cohorts of kidney transplants (GSE14346, GSE15296, and GSE46474) in peripheral blood.

	GEO Studies		
Target Genes	GSE14346 logFC	GSE15296 logFC	GSE46474 logFC
	NAR/AR(28/31)	NAR/AR (24/51)	NAR/AR (20/20)
*ABHD12*	−0.15	−0.39 (*)	0.24
*ACO1*	−0.41 (**)	−0.21 (*)	−0.03
*ATP9A*	−0.09	0.21 (*)	0.14
*AZIN1*	−0.68 (**)	−0.74 (***)	0.11
*CDKN1B*	−0.67 (***)	−0.43 (*)	0.14
*CHD2*	−0.84 (**)	0.93 (**)	−0.05
*FAM46C*	0.44	−0.53	−0.11
*LDLR*	−0.33 (*)	0.07	0.13
*MBD6*	0.45 (**)	0.34 (*)	0.39
*MDM4*	0.21	−0.39	−0.23
*MESDC2*	−1.02 (***)	−0.37 (*)	−0.12
*MLXIP*	0.31	0.28 (*)	−0.20
*MTCH2*	−0.72 (***)	−0.47 (**)	−0.11
*NAA38*	−0.45 (*)	0.28	0.23
*NBEAL1*	0.44 (*)	0.15	−0.07
*PDE7A*	−1.16 (***)	−0.86 (**)	−0.19
*PERP*	−0.83 (**)	0.21 (*)	−0.04
*SAR1A*	−0.22	−0.22	0.19
*SMC3*	−0.04	−0.69 (***)	0.21
*SOGA3*	−0.16	0.07	−0.06
*SP1*	0.27	−0.86 (***)	0.21
*VTI1A*	−0.46 (*)	0.62 (***)	0.03

AR; Acute rejection, NAR; Non-Acute Rejection. Positive fold-change values indicate an increase in the AR group, and negative values indicate an increased NAR group. Statistical significance comparisons are indicated in bold. logFC (fold-change). *p*-values obtained directly from the GEO2R web tool. FDR values < 0.05 were considered statistically significant. * FDR < 0.05; ** FDR < 0.01; *** FDR < 0.001.

## Data Availability

The study did not report any data.

## Supplementary Materials

The following are available online at https://www.mdpi.com/article/10.3390/jcm10132748/s1. Table S1: Cohorts of kidney transplants were used to compare target genes’ expression levels in peripheral blood, Table S2: Relation of genes interacts with MBD6 in three cohorts of kidney transplants (GSE14346, GSE15296, and GSE46474) in peripheral blood.

## References

[1] Singh N., Samant H., Hawxby A., Samaniego M.D. (2019). Biomarkers of rejection in kidney transplantation. Curr. Opin. Organ Transplant..

[2] When a Transplant Fails|National Kidney Foundation. https://www.kidney.org/transplantation/transaction/TC/summer09/TCsm09_TransplantFails.

[3] Friedewald J., Abecassis M., Kurian S. (2019). Gene expression biomarkers for kidney transplant rejection—The entire landscape. EBioMedicine.

[4] O’Callaghan J.M., Knight S.R. (2019). Noninvasive biomarkers in monitoring kidney allograft health. Curr. Opin. Organ Transplant..

[5] Bartel D.P. (2004). MicroRNAs: Genomics, Biogenesis, Mechanism, and Function. Cell.

[6] Wahid F., Shehzad A., Khan T., Kim Y.Y. (2010). MicroRNAs: Synthesis, mechanism, function, and recent clinical trials. Biochim. Biophys. Acta-Mol. Cell Res..

[7] Li S.C., Pan C.Y., Lin W.C. (2006). Bioinformatic discovery of microRNA precursors from human ESTs and introns. BMC Genom..

[8] Margulies M., Egholm M., Altman W.E., Attiya S., Bader J.S., Bemben L.A., Berka J., Braverman M.S., Chen Y.J., Chen Z. (2005). Genome sequencing in microfabricated high-density picolitre reactors. Nature.

[9] Gimondi S., Dugo M., Vendramin A., Bermema A., Biancon G., Cavané A., Corradini P., Carniti C. (2016). Circulating miRNA panel for prediction of acute graft-versus-host disease in lymphoma patients undergoing matched unrelated hematopoietic stem cell transplantation. Exp. Hematol..

[10] Van Huyen J.P.D., Tible M., Gay A., Guillemain R., Aubert O., Varnous S., Iserin F., Rouvier P., François A., Vernerey D. (2014). MicroRNAs as non-invasive biomarkers of heart transplant rejection. Eur. Heart J..

[11] Amarilyo G., La Cava A. (2012). MiRNA in systemic lupus erythematosus. Clin. Immunol..

[12] Ratert N., Meyer H.A., Jung M., Lioudmer P., Mollenkopf H.J., Wagner I., Miller K., Kilic E., Erbersdobler A., Weikert S. (2013). MiRNA profiling identifies candidate mirnas for bladder cancer diagnosis and clinical outcome. J. Mol. Diagn..

[13] Basso K., Sumazin P., Morozov P., Schneider C., Maute R.L., Kitagawa Y., Mandelbaum J., Haddad J., Chen C.Z., Califano A. (2009). Identification of the Human Mature B Cell miRNome. Immunity.

[14] Tan L.P., Wang M., Robertus J.L., Schakel R.N., Gibcus J.H., Diepstra A., Harms G., Peh S.C., Reijmers R.M., Pals S.T. (2009). MiRNA profiling of B-cell subsets: Specific miRNA profile for germinal center B cells with variation between centroblasts and centrocytes. Lab. Investig..

[15] Xiao C., Calado D.P., Galler G., Thai T.H., Patterson H.C., Wang J., Rajewsky N., Bender T.P., Rajewsky K. (2007). MiR-150 Controls B Cell Differentiation by Targeting the Transcription Factor c-Myb. Cell.

[16] Vigorito E., Perks K.L., Abreu-Goodger C., Bunting S., Xiang Z., Kohlhaas S., Das P.P., Miska E.A., Rodriguez A., Bradley A. (2007). microRNA-155 Regulates the Generation of Immunoglobulin Class-Switched Plasma Cells. Immunity.

[17] Danger R., Pallier A., Giral M., Martínez-Llordella M., Lozano J.J., Degauque N., Sanchez-Fueyo A., Soulillou J.P., Brouard S. (2012). Upregulation of miR-142-3p in peripheral blood mononuclear cells of operationally tolerant patients with a renal transplant. J. Am. Soc. Nephrol..

[18] Anglicheau D., Sharma V.K., Ding R., Hummel A., Snopkowski C., Dadhania D., Seshan S.V., Suthanthiran M. (2009). MicroRNA expression profiles predictive of human renal allograft status. Proc. Natl. Acad. Sci. USA.

[19] Khalid U., Newbury L.J., Simpson K., Jenkins R.H., Bowen T., Bates L., Sheerin N.S., Chavez R., Fraser D.J. (2019). A urinary microRNA panel that is an early predictive biomarker of delayed graft function following kidney transplantation. Sci. Rep..

[20] Levey A.S., Eckardt K.U., Tsukamoto Y., Levin A., Coresh J., Rossert J., De Zeeuw D., Hostetter T.H., Lameire N., Eknoyan G. (2005). Definition and classification of chronic kidney disease: A position statement from Kidney Disease: Improving Global Outcomes (KDIGO)z. Kidney Int..

[21] Levey A.S., Coresh J., Balk E., Kausz A.T., Levin A., Steffes M.W., Hogg R.J., Perrone R.D., Lau J., Eknoyan G. (2003). National Kidney Foundation Practice Guidelines for Chronic Kidney Disease: Evaluation, Classification, and Stratification. Ann. Intern. Med..

[22] Mark P.B., Petrie C.J., Jardine A.G. (2007). Diagnostic, Prognostic, And therapeutic implications of brain natriuretic peptide in dialysis and nondialysis-dependent chronic renal failure. Semin. Dial..

[23] Tabrizi R., Zolala F., Nasirian M., Baneshi M.R., Etminan A., Sekhavati E., Khodadost M., Haghdoost A.A. (2016). Estimation of the prevalence of chronic kidney disease: The results of a model based estimation in Kerman, Iran. Med. J. Islam. Repub. Iran.

[24] Haas M., Loupy A., Lefaucheur C., Roufosse C., Glotz D., Seron D., Nankivell B.J., Halloran P.F., Colvin R.B., Akalin E. (2018). The Banff 2017 Kidney Meeting Report: Revised diagnostic criteria for chronic active T cell–mediated rejection, antibody-mediated rejection, and prospects for integrative endpoints for next-generation clinical trials. Am. J. Transplant..

[25] Cohen D., Colvin R.B., Daha M.R., Drachenberg C.B., Haas M., Nickeleit V., Salmon J.E., Sis B., Zhao M.H., Bruijn J.A. (2012). Pros and cons for C4d as a biomarker. Kidney Int..

[26] DIANA TOOLS. http://diana.imis.athena-innovation.gr/DianaTools/index.php.

[27] TargetScanHuman 7.2. http://www.targetscan.org/vert_72/.

[28] GEO Accession Viewer. https://www.ncbi.nlm.nih.gov/geo/query/acc.cgi?acc=GSE115816.

[29] Li L., Khatri P., Sigdel T.K., Tran T., Ying L., Vitalone M.J., Chen A., Hsieh S., Dai H., Zhang M. (2012). A peripheral blood diagnostic test for acute rejection in renal transplantation. Am. J. Transplant..

[30] Kurian S.M., Williams A.N., Gelbart T., Campbell D., Mondala T.S., Head S.R., Horvath S., Gaber L., Thompson R., Whisenant T. (2014). Molecular classifiers for acute kidney transplant rejection in peripheral blood by whole genome gene expression profiling. Am. J. Transplant..

[31] Günther O.P., Shin H., Ng R.T., McMaster W.R., McManus B.M., Keown P.A., Tebbutt S.J., Lê Cao K.A. (2014). Novel multivariate methods for integration of genomics and proteomics data: Applications in a kidney transplant rejection study. Omi. A J. Integr. Biol..

[32] Liao Y., Wang J., Jaehnig E.J., Shi Z., Zhang B. (2019). WebGestalt 2019: Gene set analysis toolkit with revamped UIs and APIs. Nucleic Acids Res..

[33] Kanehisa M., Goto S. (2000). KEGG: Kyoto Encyclopedia of Genes and Genomes. Nucleic Acids Res..

[34] Haynes W. (2013). Encyclopedia of Systems Biology, Benjamini—Hochberg Method.

[35] Home-GEO-NCBI. https://www.ncbi.nlm.nih.gov/geo/.

[36] Jonas S., Izaurralde E. (2015). Towards a molecular understanding of microRNA-mediated gene silencing. Nat. Rev. Genet..

[37] Matz M., Heinrich F., Lorkowski C., Wu K., Klotsche J., Zhang Q., Lachmann N., Durek P., Budde K., Mashreghi M.-F. (2018). MicroRNA regulation in blood cells of renal transplanted patients with interstitial fibrosis/tubular atrophy and antibody-mediated rejection. PLoS ONE.

[38] Karytinos A., Forneris F., Profumo A., Ciossani G., Battaglioli E., Binda C., Mattevi A. (2009). A novel mammalian flavin-dependent histone demethylase. J. Biol. Chem..

[39] Backes C., Meese E., Keller A. (2016). Specific miRNA Disease Biomarkers in Blood, Serum and Plasma: Challenges and Prospects. Mol. Diagn. Ther..

[40] Khan Z., Suthanthiran M., Muthukumar T. (2019). MicroRNAs and Transplantation. Clin. Lab. Med..

[41] Smith N.L., Wissink E.M., Grimson A., Rudd B.D. (2015). MiR-150 Regulates Differentiation and Cytolytic Effector Function in CD8+ T cells. Sci. Rep..

[42] Wang F., Ren X., Zhang X. (2015). Role of microRNA-150 in solid tumors. Oncol. Lett..

[43] Zhang Z., Wang J., Li J., Wang X., Song W. (2018). MicroRNA-150 promotes cell proliferation, migration, and invasion of cervical cancer through targeting PDCD4. Biomed. Pharmacother..

[44] Watanabe A., Tagawa H., Yamashita J., Teshima K., Nara M., Iwamoto K., Kume M., Kameoka Y., Takahashi N., Nakagawa T. (2011). The role of microRNA-150 as a tumor suppressor in malignant lymphoma. Leukemia.

[45] Hippen K.L., Loschi M., Nicholls J., MacDonald K.P.A., Blazar B.R. (2018). Effects of MicroRNA on Regulatory T Cells and Implications for Adoptive Cellular Therapy to Ameliorate Graft-versus-Host Disease. Front. Immunol..

[46] de Candia P., Torri A., Gorletta T., Fedeli M., Bulgheroni E., Cheroni C., Marabita F., Crosti M., Moro M., Pariani E. (2013). Intracellular Modulation, Extracellular Disposal and Serum Increase of MiR-150 Mark Lymphocyte Activation. PLoS ONE.

[47] Wilflingseder J., Regele H., Perco P., Kainz A., Soleiman A., Mühlbacher F., Mayer B., Oberbauer R. (2013). MiRNA profiling discriminates types of rejection and injury in human renal allografts. Transplantation.

[48] Soltaninejad E., Nicknam M.H., Nafar M., Ahmadpoor P., Pourrezagholi F., Sharbafi M.H., Hosseinzadeh M., Foroughi F., Yekaninejad M.S., Bahrami T. (2015). Differential expression of microRNAs in renal transplant patients with acute T-cell mediated rejection. Transpl. Immunol..

[49] Cron M.A., Maillard S., Truffault F., Gualeni A.V., Gloghini A., Fadel E., Guihaire J., Behin A., Berrih-Aknin S., Le Panse R. (2019). Causes and Consequences of miR-150-5p Dysregulation in Myasthenia Gravis. Front. Immunol..

[50] Roat R., Hossain M.M., Christopherson J., Free C., Guay C., Regazzi R., Guo Z. (2019). Circulating miRNA-150-5p is associated with immune-mediated early β-cell loss in a humanized mouse model. Xenotransplantation.

[51] Guo X., Zhu Y., Hong X., Zhang M., Qiu X., Wang Z., Qi Z., Hong X. (2017). miR-181d and c-myc-mediated inhibition of CRY2 and FBXL3 reprograms metabolism in colorectal cancer. Cell Death Dis..

[52] Liu F., Lou Y.-L., Wu J., Ruan Q.-F., Xie A., Guo F., Cui S.-P., Deng Z.-F., Wang Y. (2012). Upregulation of MicroRNA-210 Regulates Renal Angiogenesis Mediated by Activation of VEGF Signaling Pathway under Ischemia/Perfusion Injury in vivo and in vitro. Kidney Blood Press. Res..

[53] Vitalone M.J., Sigdel T.K., Salomonis N., Sarwal R.D., Hsieh S.C., Sarwal M.M. (2015). Transcriptional Perturbations in Graft Rejection. Transplantation.

[54] Yang H., Zhang J., Li J., Zhao F., Shen Y., Xing X. (2018). Overexpression of miR-574-3p suppresses proliferation and induces apoptosis of chronic myeloid leukemia cells via targeting IL6/JAK/STAT3 pathway. Exp. Ther. Med..

[55] Jung J.S., Jee M.K., Cho H.T., Choi J.I., Im Y.B., Kwon O.H., Kang S.K. (2013). MBD6 is a direct target of Oct4 and controls the stemness and differentiation of adipose tissue-derived stem cells. Cell. Mol. Life Sci..

[56] Saito Y., Miyagawa Y., Onda K., Nakajima H., Sato B., Horiuchi Y., Okita H., Katagiri Y.U., Saito M., Shimizu T. (2008). B-cell-activating factor inhibits CD20-mediated and B-cell receptor-mediated apoptosis in human B cells. Immunology.

[57] Teruel-Montoya R., Kong X., Abraham S., Ma L., Kunapuli S.P., Holinstat M., Shaw C.A., McKenzie S.E., Edelstein L.C., Bray P.F. (2014). MicroRNA expression differences in human hematopoietic cell lineages enable regulated transgene expression. PLoS ONE.

[58] Pritchard C.C., Kroh E., Wood B., Arroyo J.D., Dougherty K.J., Miyaji M.M., Tait J.F., Tewari M. (2012). Blood cell origin of circulating microRNAs: A cautionary note for cancer biomarker studies. Cancer Prev. Res..

[59] Juzenas S., Venkatesh G., Hübenthal M., Hoeppner M.P., Du Z.G., Paulsen M., Rosenstiel P., Senger P., Hofmann-Apitius M., Keller A. (2017). A comprehensive, cell specific microRNA catalogue of human peripheral blood. Nucleic Acids Res..

[60] Möhnle P., Hirschberger S., Hinske L.C., Briegel J., Hübner M., Weis S., Dimopoulos G., Bauer M., Giamarellos-Bourboulis E.J., Kreth S. (2018). MicroRNAs 143 and 150 in whole blood enable detection of T-cell immunoparalysis in sepsis. Mol. Med..

