# Supplementary material for: MicroRNA Expression Changes in Kidney Transplant: Diagnostic Efficacy of miR-150-5p as Potential Rejection Biomarker, Pilot Study
Source: J Clin Med. 2021 Jun 22;10(13):2748. doi: 10.3390/jcm10132748 (PMC8268834; doi:10.3390/jcm10132748)
Supplement: Supplementary file 1 [file jcm-10-02748-s001.zip › jcm-1247477-supplementary.pdf]

Supplementary materials

Table S1. Cohorts of kidney transplants were used to compare target genes' expression levels in peripheral blood.

GEO accession	Summary	Over all design	Reference
GSE115816	MicroRNAs of whole blood cells isolated from patients after kidney transplantation with stable graft function (SGF), antibody-mediated rejection (ABMR), and T cell-mediated rejection (TCMR).	Whole blood cells were isolated from six patients with stable graft function, six patients with histologically verified antibody-mediated graft rejection episodes, and four histologically verified T cell-mediated graft rejection after kidney transplantation.	[28]
GSE14346	Authors examined transcriptional profiles from 3 different microarray platforms, across 103 peripheral blood samples with and without acute rejection, to find a critical gene-set for the diagnosis of acute renal rejection that matched biopsy diagnosis, irrespective of patient demographics, clinical confounders, concomitant infection, immunosuppression usage or sample processing methods.	Cross-sectional microarray analysis of 103 matched peripheral blood samples collected at a single time point, timed with a biopsy where acute rejection was either confirmed as present (60 AR samples) or absent (62 STA samples).	[29]
GSE15296	The authors used whole-genome expression profiling of peripheral blood samples from 51 patients with biopsy-proven acute kidney transplant rejection and 24 patients with excellent function and biopsy-proven normal transplant histology.	Microarray profiles of peripheral blood from 51 biopsy-proven acute kidney rejection (AR) and 24 well-functioning kidney transplants were randomized and compared using class comparisons, network, and biological function analyses.	[30]
GSE46474	Authors study gene expression changes using whole genome microarray analysis of peripheral blood from subjects with acute rejection (n = 20) and non-rejecting controls (n = 20) to obtain insight into the molecular and biological causation of acute renal allograft rejection when combined with proteomics (iTRAQ) data for the same patients/time-points.	40 Samples	[31]

Table S2. Relation of genes interacts with MBD6 in three cohorts of kidney transplants (GSE14346, GSE15296, and GSE46474) in peripheral blood.

	**GSE14346**		**GSE15296**		**GSE46474**	
	**logFC**	**FDR**	**logFC**	**FDR**	**logFC**	**FDR**
**KDM1B**	−0.140	ns	−0.23	ns	0.471	ns
**ASXL2**	−0.100	ns	−0.06	ns	0.091	ns
**BAP1**	−0.065	ns	−0.2	ns	−0.030	ns
**FOXK2**	0.057	ns	−0.12	ns	−0.020	ns
**HCFC1**	−0.624	0.007	0.05	ns	0.050	ns
**OGT**	0.054	ns	−0.559	0.003	0.120	ns
**WDR5**	0.041	ns	−0.125	ns	−0.080	ns
**SETD1A**	−0.705	0.011	0.08	ns	0.020	ns
**ASH2L**	−0.290	0.015	−0.17	ns	0.008	ns
**RBBP5**	0.139	ns	0.139	ns	−0.300	ns

ns: Not statistically significant. logFC, Log Fold Change. FDR, False Discovery Rate. LogFC values are ratios (gene expression in the AR group/gene expression in the NAR group). Negative logFC values indicate that in this cohort, that gene is increased in the NAR group, while if it gives positive values, it indicates that the gene expression is increased in the AR group.
